# Supplementary material for: The interactome of intact mitochondria by cross-linking mass spectrometry provides evidence for coexisting respiratory supercomplexes
Source: Mol Cell Proteomics. 2017 Dec 8;17(2):216–32. doi: 10.1074/mcp.RA117.000470 (PMC5795388; doi:10.1074/mcp.RA117.000470)
Supplement: Supplemental Data [file supp_RA117.000470_133922_0_supp_23978_fzffwf.zip › spectra_annotation/mito_spectra_annotation/53-1-55-1-11-1.pdf]

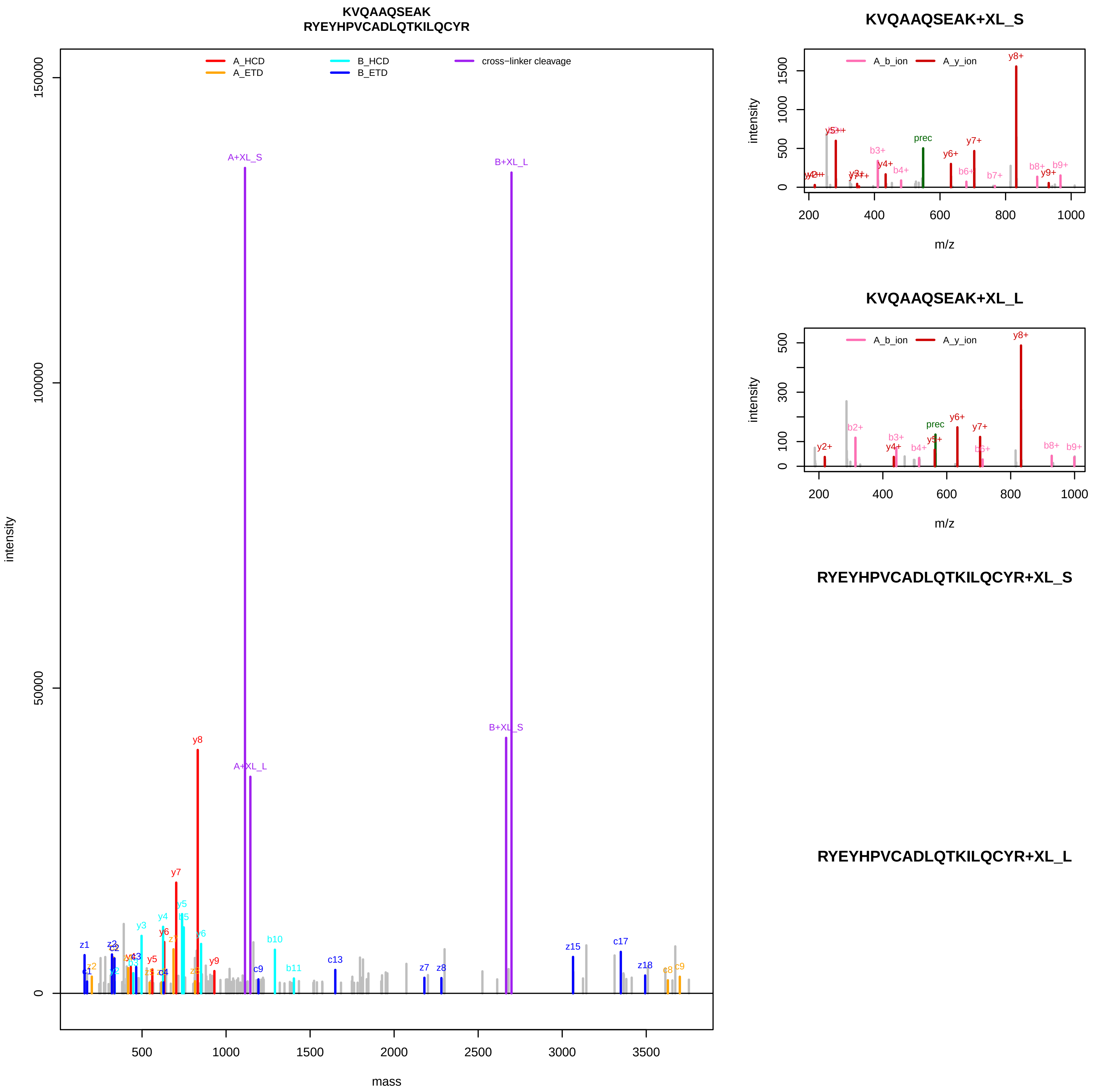
KVQAAQSEAK
RYEYHPVCADLQTKILQCYR
A_HCD
A_ETD
B_HCD
B_ETD
cross−linker cleavage
A+XL_S
B+XL_L
B+XL_S
A+XL_L
y8
y7
y9
y5
y6
y3
y4
b5
y6
b10
b11
c9
c13
z1
c1
z2
c2
c3
b3
c4
z7
z8
z15
c17
z18
c8
c9
0
50000
100000
150000
500
1000
1500
2000
2500
3000
3500
intensity
mass
KVQAAQSEAK+XL_S
A_b_ion
A_y_ion
y8+
y5++
prec
y7+
y6+
b3+
y4+
b4+
b6+
b7+
b8+
b9+
y9+
0
500
1000
1500
200
400
600
800
1000
intensity
m/z
KVQAAQSEAK+XL_L
A_b_ion
A_y_ion
y8+
b2+
prec
y6+
y7+
y2+
b3+
y5+
y4+
b4+
b6+
b8+
b9+
0
100
300
500
200
400
600
800
1000
intensity
m/z
RYEYHPVCADLQTKILQCYR+XL_S
RYEYHPVCADLQTKILQCYR+XL_L
